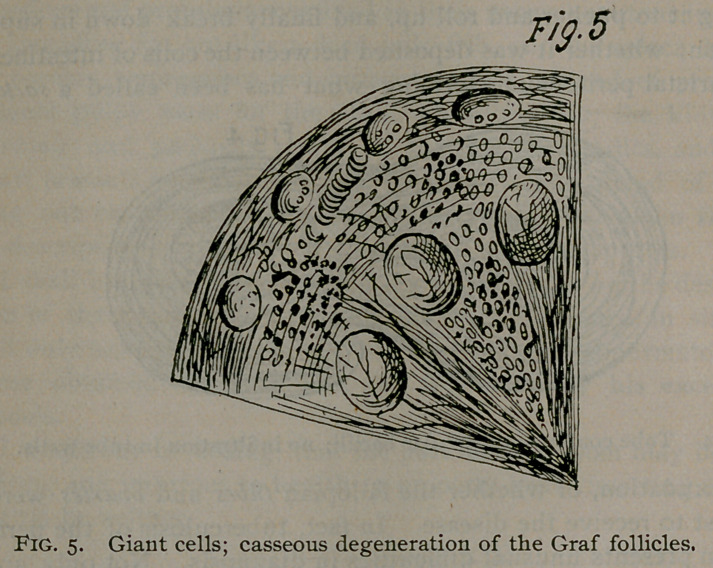# Tubercular Peritonitis

**Published:** 1892-06

**Authors:** T. J. Crofford

**Affiliations:** Memphis, Tenn., Gynecologist to St. Joseph’s Hospital, Memphis Sanitarium, Etc.


For Daniel's Texas Medical Journal.

### TUBERCULAR PERITONITIS.

BY T. J. CROFFORD, M. D., MEMPHIS, TENN.,
Gynecologist to St. Joseph's Hospital, Memphis Sanitarium, Etc.

CASE: Mrs. C., aged 23, married one year, no children, was brought to me on 7th Dec., 1890, with a history of an abdominal inflammation. The abdomen now presented a nodulated enlargement the size of a large cocoanut, midway between the umbilicus and pubes. Several members of her family had died with pulmonary tuberculosis. This, together with her history and symptoms, and the microscopic appearance upon section, make it out clearly a case of tubercular disease. A full, free incision was made down to the purulent collection. Nothing was removed. The pus was evacuated and the pocket kept packed with gauze. She improved for a short while, when a new point of invasion manifested itself. This was in due time similarly dealt with, then another and another until some five, six or seven points had been opened. It has been now some fifteen months since the last incision was made, and she writes me that she does not remember to have ever been so well as now, nor does she know any one else who enjoys better health than she.

I am unable to say whether the primary invasion was *omental*, causing it to pucker and roll up, and finally break down in suppuration; whether it was deposited between the coils of intestines and parietal peritoneum, forming what has been called a *sacu-*

FIG. 4. Tube contents; tubercular bacilli; no infiltration in tube walls.

*lated* exudation, or whether the fallopian *tubes* and *ovaries* were the first to receive the disease. In fact, tuberculosis of the peritoneum presents unusual difficulties in diagnosis. Not only are we to distinguish from ovarian, uterine and other tumors, but as to the special kind of the tuberculosis existing, and the organ or organs involved. To such an extent are these difficulties present that the very elect in gynecology are deceived. Perhaps surprises are more frequent here than in other abdominal diseases. In 96 cases of tuberculosis in which laparotomy was performed, 37 had been diagnosed tumor, ovarian or otherwise, tuberculosis not being suspected.

CASE 2. Mrs. R., multipara, aged 28. One year ago last January, abdominal section was performed by a prominent gynecologist. Tuberculosis peritonitis was diagnosed. The incision was at once closed. Nothing was removed on account of the nature of the disease and the adhesions present. The condition of the patient grew gradually worse, the abdomen filled with serum and the situation was very unenviable, although the strength and general appearance kept up remarkably well. She was brought to me on January 5th, 1892, the abdomen was reopened by making an incision just above the former one and extending down almost through the old one. After evacuating several gallons of serous accumulation from the cavity, the tuberculosis deposits which were scattered almost entirely throughout the abdominal cavity were plainly to be seen. The excessively tuberculous tubes and ovaries and intestines which surrounded them, were adhered to each other and to the uterus to the extent of constituting a rounded mass which was also adhered to the

Fig. 5

FIG. 5. Giant cells; casseous degeneration of the Graf follicles.

abdomen along the line of the old incision. The task of unraveling this mass took upwards of an hour, and in four places the peritoneal and muscular coats were stripped off the small intestines, which were at once repaired by fine silk and a cambric needle. After removal of the tubes and ovaries the abdomen was carefully but thoroughly washed out, and adhesions between coils of intestines broken up. A glass drainage tube was placed down posterior to the uterus and the abdomen closed. The case ran a smooth course from this time on, and she went to her home, 150 miles distant, on Feb. 4th.

MICROSCOPIST'S REPORT.

"DR. T. J. CROFFORD:—The following is my report: The specimen consists of tubes and ovaries and part of broad ligaments. Right ovary is about the size of a pigeon egg and covered with milliary tubercles. The left ovary is somewhat smaller and spherical. The tubes are tortuous, hard, thick, very much dilated and bound to the broad ligaments by false membrane; the whole mass is covered with milliary tubercles.

"On section I find the right ovary to contain about a teaspoonful of thick, yellow pus, the left ovary is also filled with pus. Contents of tubes are pus and tubercular debris. Inoculations of glycerine gelatine tubes made from pieces of ovaries and tubes, showed after two weeks at a temperature of 38° C., growths of whitish scales, which upon examination presented the characteristics of the bacillus tuberculosis. Sections stained after Ehrlich's and Weigert's method, and examined under the microscope, show giant tubercle cells on the margin of the ovaries, and numerous bacilli throughout the specimen.

"M. B. HERMAN, M. D."

Since she has been home her condition has been variable. At one time she will write that she is rapidly being restored to health, then again that her condition is not so favorable. Whether or not she will perfectly recover is a question, yet this much is evident, that tubercular peritonitis is an operatable case, and if taken in time and the diseased structures be thoroughly removed and the abdomen thoroughly irrigated, can be cured. Let us hope that these conditions may yet become curable by the surgeon, like many other conditions formerly regarded as hopeless are now perfectly relieved by operative interference. We are in the formative stage of tubercular peritonitis. The lines which are to guide us have not been laid down, yet, but are to be determined in the future. A literature is yet to be created. What has been and what is being done in the way of operations for relief and the results therefrom is the desirable thing to be known. In my opinion, the honest report of a single case will outweigh all the theory and speculation imaginable.

CONCLUSIONS.

1. Tubercular peritonitis is an operatable case.
2. An early operation is of greatest value.
3. The chronic variety offers the best indications for surgical interference.
4. When the primary deposit is in the tubes, (which Winckel declares to be in fifty per cent. of the cases), on early salpingotomy will cure the disease.
5. Operations later in the disease will frequently prolong life, and possibly cure.

## Figures and Tables

**Fig. 4. f1:**
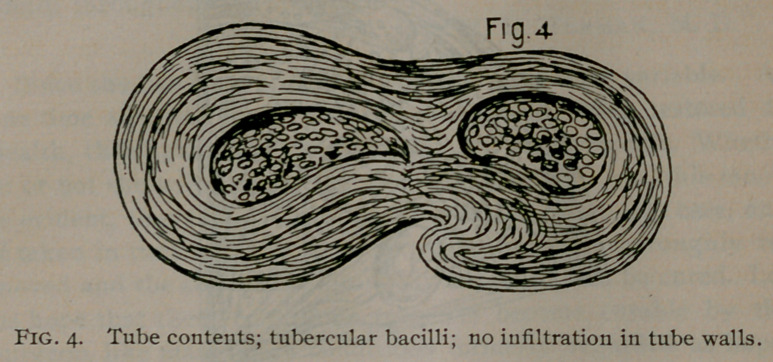


**Fig. 5. f2:**